# Initial phantom studies for an office-based low-field MR system for prostate biopsy

**DOI:** 10.1007/s11548-021-02364-7

**Published:** 2021-04-23

**Authors:** Selin Chiragzada, Eva Hellman, Duncan Michael, Ram Narayanan, Aleksandar Nacev, Dinesh Kumar

**Affiliations:** Promaxo Inc, Oakland, CA 94607 USA

**Keywords:** Targeted prostate biopsy, Low-field MRI, Office-based MRI, Navigation accuracy, Prostate cancer, MR-guided biopsy

## Abstract

**Purpose:**

Prostate cancer is the second most prevalent cancer in US men, with about 192,000 new cases and 33,000 deaths predicted for 2020. With only a 31% 5-year survival rate for patients with an initial diagnosis of stage-four prostate cancer, the necessity for early screening and diagnosis is clear. In this paper, we present navigation accuracy results for Promaxo’s MR system intended to be used in a physician’s office for image-guided transperineal prostate biopsy.

**Methods:**

The office-based low-field MR system was used to acquire images of prostate phantoms with needles inserted through a transperineal template. Coordinates of the estimated sample core locations in the office-based MR system were compared to ground truth needle coordinates identified in a 1.5T external reference scan. The error was measured as the distance between the planned target and the ground truth core center and as the shortest perpendicular distance between the planned target and the ground truth trajectory of the whole core.

**Results:**

The average error between the planned target and the ground truth core center was 2.57 ± 1.02 mm, [1.93–3.21] 95% CI. The average error between the planned target to the actual core segment was 2.05 ± 1.24 mm, [1.53–2.56] 95% CI.

**Conclusion:**

The average navigation errors were below the clinically significant threshold of 5 mm. The initial phantom results demonstrate the feasibility of the office-based system for prostate biopsy.

## Introduction

Prostate cancer is the second most prevalent cancer in US men. For the year 2020, it is predicted that there will be about 192,000 new cases and 33,000 deaths from prostate cancer [1]. The 5-year survival rate for patients initially diagnosed with local or regional prostate cancer is almost 100% but drops to 31% for those with an initial diagnosis of prostate cancer that has metastasized (stage four) [2]. The necessity for early screening and diagnosis is clear.

The most common technique and the standard of care for prostate cancer diagnosis is a standalone systematic transrectal ultrasound-guided biopsy (TRUS). These biopsies are performed if the patient’s blood prostate-specific antigen (PSA) levels are found to be elevated or if abnormalities are found in a digital rectal examination [3]. In most cases, an ultrasound-guided biopsy is performed transrectally. Standard TRUS biopsies suffer from a few limitations. TRUS usually targets the periphery of the prostate; however, 30–40% of prostate cancer is found anteriorly, in the midline transition zone or in the apex [4]. In addition, since the biopsy needle passes through the rectum, contaminations leading to infection and even sepsis may occur [5]. An alternative to the transrectal approach is the transperineal approach (TPUS), which allows for easier access to all parts of the prostate and avoids contamination by the rectum, therefore resulting in greater accuracy and lower infection rates. However, the random ‘blind’ sampling employed in standard TRUS and TPUS techniques can miss cancerous lesions [6] and only 17–57% of the lesions visible on ultrasound are malignant [7]. For TRUS, first-time biopsy diagnostic yields have been reported to be between 40 and 50% [8].

Magnetic resonance imaging (MRI) is a common imaging modality used in many medical practices for diagnosis, including the field of prostate cancer care [9]. The peripheral zone (PZ) of the prostate, which is the region of the gland where adenocarcinoma is most common [10], is clearly delineated on prostate MR images. As a result, MR imaging can be used to determine next steps for a patient with elevated PSA levels [11]. In recent years, techniques such as MRI-ultrasound fusion have emerged as a way to target lesions more precisely [12, 13], compared to the traditional systematic transrectal and transperineal ultrasound biopsy techniques. These targeted MRI-based approaches have been reported to show improvement in rates of cancer detection compared to a systematic 12-core biopsy [14]. However, such techniques may have limitations in accounting for large gland deformation as a result of application of transrectal ultrasound transducers [15, 16]. For example, Hu et al. [16] estimated the registration errors to be more than 6 mm on a large number of patient data for segmentation-based automatic registration methods commonly employed by fusion systems.

**Fig. 1:**
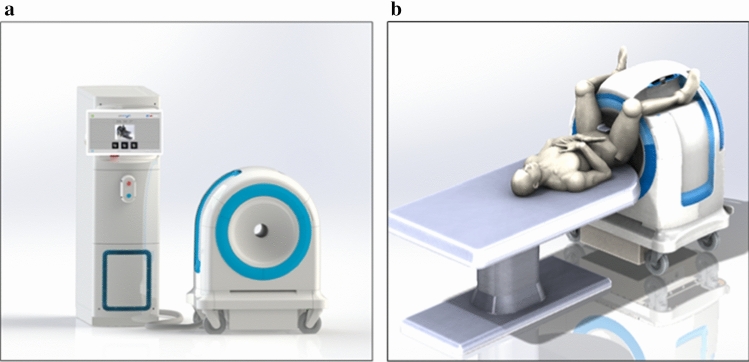
**a** The low-field MRI system. The auxiliary cart (left) houses the electrical and electronic components, such as the computer, programmable logic controller, power distribution unit and amplifiers. The magnet cart (right) houses the magnet, gradient coils and transmission coil and attaches to the receive coil. **b** The scanning position is a lithotomy position with the patient’s legs around the magnet cart

The Promaxo MRI System (Fig. 1a) is a single-sided office-based low-field (0.066 T) MRI system intended to be used for MR image-guided transperineal prostate biopsy in a urologist’s office [17, 18]. The system is designed to overcome traditional barriers associated with performing prostate biopsies under direct MR guidance. Its compact design and low magnetic field result in a limited fringe field, low energy utilization and absence of any hazardous materials such as cryogens. As a result, the system is ideally suited for office-based procedures without requiring any significant facility upgrades.

The office-based system produces transverse, sagittal and coronal cross-sectional images to display the prostate and adjoining tissues. The patient is positioned such that the region to be imaged is within the imaging field of view (Fig. 1b), and the user performs the scan using the touchscreen interface. The system displays the reconstructed images on a monitor.

The MR system includes a navigation software to plan targets and direct the biopsy needle to the location of interest through a transperineal template (a mechanical structure with holes used as a needle guide). The study assesses the navigation accuracy when the system is used for guidance. In PI-RADS™ v2.1, a clinically significant cancer is defined as having a volume greater than or equal to 0.5 cm^3^ [19]. The radius of a spherical 0.5 cm^3^ tumor is about 5 mm. Therefore, 5 mm was used as the acceptance criterion for this study.

## Methods and materials

### General navigation workflow

**Fig. 2:**
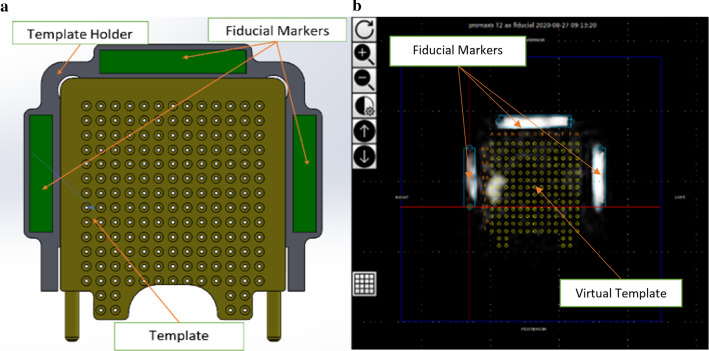
**a** The template holder is built with rectangular-shaped fiducials in a fixed position. Three fiducials are enclosed within the template holder. The template secures into place onto the template holder, and the user replaces the template between patients. **b** A screenshot of the template calibration page from the system’s GUI. The image shows the expected fiducial locations displayed as blue rectangular boxes on the left, right and top of the estimated virtual template represented in yellow. The user may scroll through the fiducial localizer scan and rotate or translate the overlay to match it to the fiducials on the image

First, a scan consisting of multiple cross-sectional images, or slices, is obtained on the office-based MR system. The user then performs template calibration by clicking on the fiducial markers on the image using the graphical user interface (GUI). The purpose of template calibration is to identify the template with reference to the image such that the needle trajectories can be accurately computed. Note that the template is usually made of plastics or metal. While plastic is invisible to MRI, metal templates are undesirable even if they are nonmagnetic due to potential field distortion. Fiducials placed on the template holders are used as points of geometric reference (Fig. 2a). The entire assembly is rigidly fixed relative to the patient. The template holder is mounted on the rigid external pelvic receive coil, which is worn by the patient during a biopsy procedure, such that the template when attached is fixed in a rigid frame of reference with respect to the patient’s perineum.

The user is trained how to select fiducials during system training. Three fiducials are shown in Fig. 2a on the left, right and above the template. Using known fiducial locations and their positions identified on the image, the system is calibrated to determine the location of the transperineal template, following which a virtual template is overlaid on the image (Fig. 2b). The study used a commercially available transperineal grid template with standard grid spacing of 5 mm. The virtual template is shown on all slices, which represents the location where a needle would be positioned if inserted through each template coordinate along a straight line. The closest template coordinate to where the user clicks to plan a target is selected as the planned target. The system displays the physical template coordinate and depth for each target. Template coordinates correspond to labeled holes on the physical template. The depth is the extent to insert the biopsy cannula such that the target will be located at the center of the core. Once the coordinates are planned, the patient is moved away from the magnet cart. The lithotomy position (e.g., with stirrups used for positioning attached to the patient bed) and relative template location are maintained, which allows the physician access to the perineum. After cannula insertion, the needle is then inserted manually through the cannula.

### Materials

#### Phantoms

**Fig. 3:**
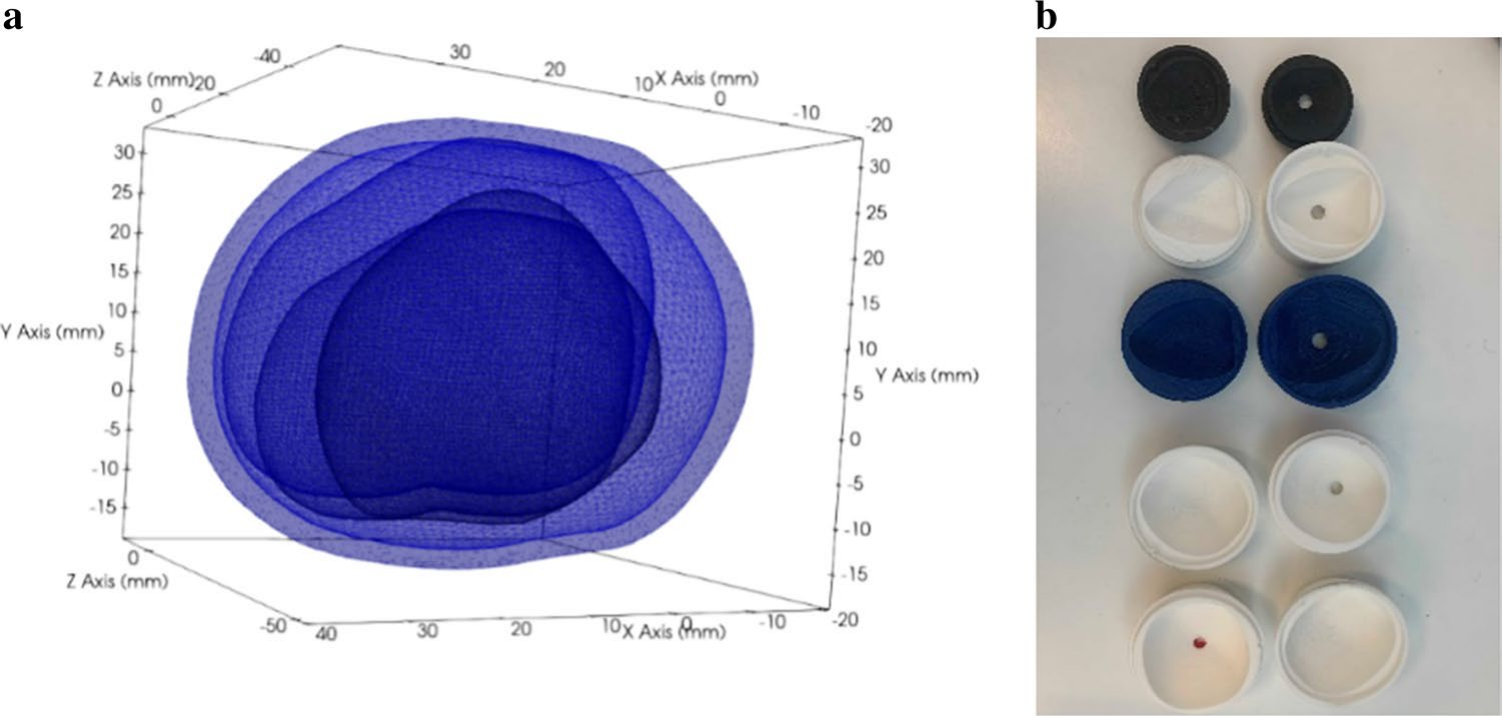
**a** The figure shows overlays of the surface models extracted from clinical MR images of the prostate and used for creating the phantom molds. The prostates have a wide range of shapes and sizes. The sizes were scaled to 25 cc, 40 cc, 60 cc, 90 cc and 120 cc, respectively. **b** Prostate molds used for the study. Top to bottom: 25 cc, 40 cc, 60 cc, 90 cc, 120 cc

The study utilizes custom-designed prostate phantoms for guidance under MRI. The phantoms are designed to mimic the shape, size and contrast characteristics of human prostate tissue. The prostate models were generated by segmenting surfaces from images from the Cancer Imaging Archive’s prostate MR studies [20] and from other MR images obtained from clinical subjects. Segmentations were performed using ITK-SNAP [21], and the segmented surfaces were smoothed using 3D Slicer [22]. Five sizes of prostate models were generated to span a range of prostate volumes [23]: 25 cc, 40 cc, 60 cc, 90 cc and 120 cc (Fig. 3a). From these models, negative molds were designed and 3D printed (Fig. 3b). The phantom consists of a prostate model encased in a background material. The prostate model was made with a mixture of a 0.021 M sodium chloride plus 3.5 mM copper sulfate solution and 6% beef gelatin. The background material was represented by a 0.05 mM manganese chloride solution and 6% beef gelatin. These solutions for the prostate and background tissue were chosen to approximate the range of T1/T2 relaxation times of human tissue. The phantom and background material were housed inside a 3D-printed box.

#### Template and fiducials

A modified off-the-shelf transperineal template was used with an off-the-shelf prostate biopsy needle kit. The template was attached to a custom 3D-printed template holder. Commercially available MR contrast gels were used as fiducial markers.

#### Other

The off-the-shelf biopsy needle kit comprises of a 15G cannula, a stiletto and a 16G biopsy gun holding a biopsy needle. The cannula is placed to the depth computed and displayed to the user by the system’s navigation software based on the desired sampling location. The cannula is initially inserted with a stiletto to create the needle track, and the stiletto is removed prior to insertion of the biopsy needle. The software takes into account the biopsy kit’s measurements, such as throw distance when the biopsy needle is inserted into the cannula and fired.

MRI scans used for ground truth measurements were acquired using a commercially available 1.5T MR scanner. MRI was chosen for ground truth measurements because needles could be easily visualized.

### Procedure

#### Office-based MR scan and needle placement

**Fig. 4:**
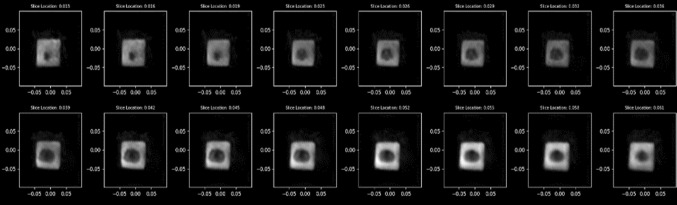
Representative volumetric axial slices of the prostate phantoms using the Promaxo system. This figure displays the individual axial slices for a T2-weighted scan taken with a pixel size of 2 mm × 2 mm in-plane resolution with 3-mm slice spacing

**Fig. 5:**
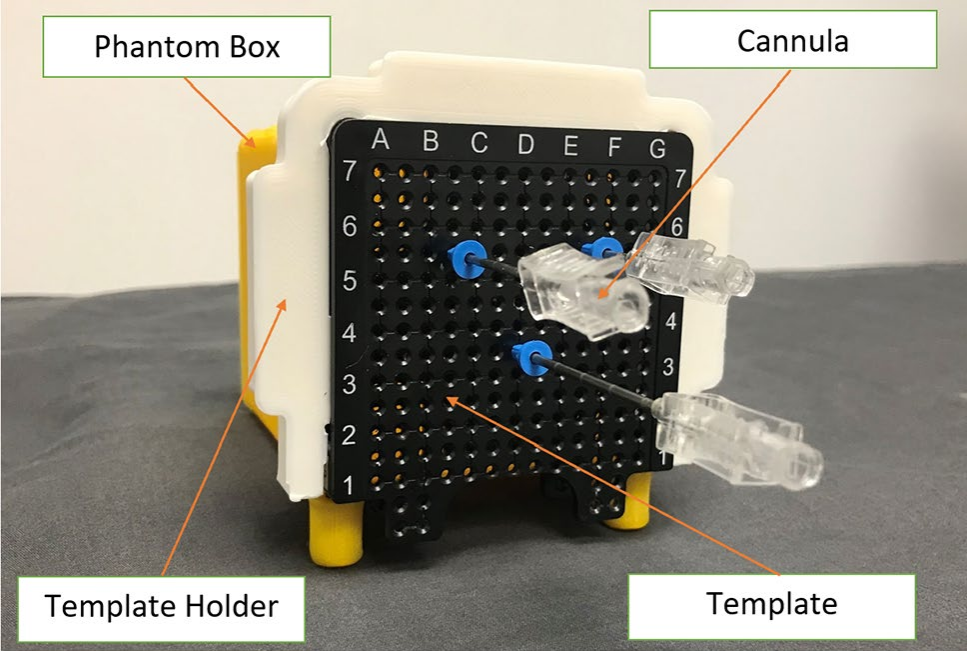
Three 15G cannulas inserted into the prostate phantom box through a transperineal template with the template holder attached at recorded coordinates and depths. A biopsy needle is inserted through each cannula in a typical procedure

A standard T2 scan of the phantom was acquired using the office-based MR system (Fig. 4). The scan also included imaging of the fiducials identifiable in MR images within the template holder. Three or more 15G needles were inserted into each of the five prostate phantoms (Fig. 5) such that target locations spanned the volume of the prostate model. The template coordinates and depths of the inserted needles were recorded.

### Ground truth determination

A 1.5T MRI scan of each phantom with the inserted cannulas was acquired at an external facility. Axial and sagittal images were acquired at an in-plane resolution of 0.23 × 0.23 mm^2^ and slice thickness of 3.48 mm each. Ground truth measurements were established by three urologists in total, where two urologists independently identified the coordinates of the needle track for each planned target. The urologists used the axial and sagittal images to identify the needle entry point and needle tip on the 1.5T image using 3D Slicer. Based on the selections, ground truth coordinates of the planned target (center of core) that would have been sampled were extrapolated.

### Projected Core Measurement

Four board-certified urologists, who were trained on the template calibration step of the navigation workflow, selected fiducial markers on the low-field image using the system’s graphical user interface. Using the calibration transform from fiducial marker selection, the template coordinate and the depth of the needle, the coordinate of each planned target was retroactively determined on the low-field image frame of reference. To compare the ground truth coordinates and the planned target coordinates, the low-field and the 1.5T images were registered such that the images were in the same frame of reference. The manual global rigid registration was performed using corners and boundaries of the box containing the phantom and object boundaries as a reference. Registration was used only as part of the experimental methodology. The registration transform was recorded.

### Analysis

**Fig. 6:**
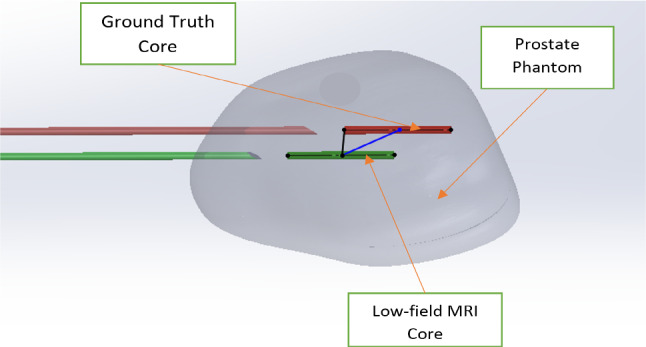
Visualization of the error measurements. The green cylinder represents the projected core from the low-field MRI system, and the red cylinder represents the ground truth core. The blue line represents the center-to-center distance between the cores, and the black line represents the shortest perpendicular distance between the planned target and the true core trajectory (point to line)

The average (mean) of the ground truth coordinates was calculated. In addition, the average distance, standard deviation and the minimum and maximum distance values between the two sets of ground truth measurements were computed. Error was assessed as i) the distance between the planned target and the center of the core (center to center) and ii) the shortest perpendicular distance between the planned target and the core segment (point to line) (Fig. 6). The shortest distance between the cores is acceptable as an error measurement because the user is intending to take the core at some place within the core representation. The average absolute error was calculated and compared to the acceptance criteria (5 mm). Standard deviation, 95% CI, minimum, maximum and interobserver variability of the error were computed.

1 $$\surd ({\left({x}_{2}-{x}_{1}\right)}^{2}+{\left({y}_{2}-{y}_{1}\right)}^{2}+{\left({z}_{2}-{z}_{1}\right)}^{2}$$

where x, y and z are coordinates of the points.

2 $$\frac{|\stackrel{-}{{M}_{0}{M}_{1}}\times \stackrel{-}{\mathrm{s}}|}{|\stackrel{-}{\mathrm{s}}|}$$

where ${M}_{0}$ represents the coordinates of the point, $s$ is the directing vector of the line, and ${M}_{1}$ represents a point on the line.

## Results

### Ground truth user variability

Table 1 summarizes the user variability of ground truth measurements of needle tip coordinates across two urologists. A total of 17 needles were inserted into the phantoms. User variability was calculated using needle tip coordinates only. We found that the average difference between the two sets of measurements was 2.32 ± 1.51 mm, with a minimum of 0.00 mm and maximum of 5.13 mm.

**Table 1:** Ground truth measurement user variability results

Ground truth user variability item	Result
Number of needles	17
Average distance between selected points across the users (mm)	2.32
Min distance between selected points across the users (mm)	0.00
Max distance between selected points across the users (mm)	5.13
Standard deviation	1.51

### Error measurements

**Fig. 7:**
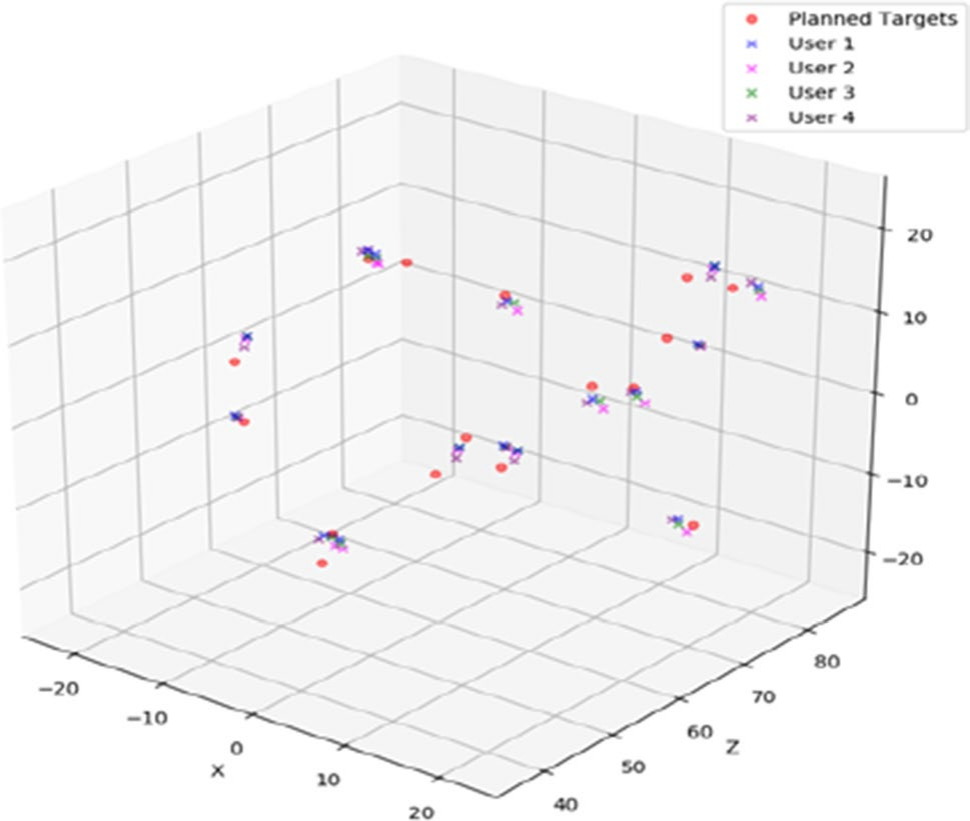
A scatter plot representation of the planned targets and the centers of the cores. The red dots represent the ground truth established by averaging the measurements made by two urologists. The crosses represent the planned targets calculated based on each physician’s template calibration

Table 2 shows the statistics for the navigation errors measured from planned target locations based on the urologists’ fiducial alignment. One of the 17 needles was excluded from the error measurement analysis because the entry point was difficult to visualize, so there were a total of 64 samples (16 needles for each of the 4 physicians). For the center-to-center error, the average was 2.57 ± 1.02 mm, 95% CI was [1.93–3.21], and interobserver variance was 0.0029 ${\mathrm{mm}}^{2}$. For the point to line error, the average was 2.05 ± 1.24 mm, 95% CI was [1.53–2.56], and interobserver variance was 0.039 ${\mathrm{mm}}^{2}$. Figure 7 is a representation of the ground truth and the targets as planned by urologists. The figure shows that the target locations were spread throughout the prostate volumes.

**Table 2:** Error measurement results

Error measurement	Planned target to center of core	Planned target to core segment
Number of samples	64	64
Average error (mm)	2.57	2.05
Min (mm)	0.93	0.20
Max (mm)	4.68	4.61
Standard deviation (mm)	1.02	1.24
95% confidence interval	[1.93–3.21]	[1.53–2.56]
Interobserver variance (mm^2^)	0.0029	0.039

## Conclusions and discussion

The Promaxo MRI System is the first single-sided low-field MRI system designed for performing image-guided procedures in office settings. Using the system’s imaging and navigation software, cannulas were inserted into prostate phantoms. The average navigation errors were found to be less than 3 mm and were therefore within the limit of less than 5 mm unsigned average error and acceptable for prostate cancer biopsy. The reported error represents contributions from the cumulative errors of fiducial calibration and image registration as well as the 3.0 mm z-resolution of the MR images used for the ground truth. Needle deflection was also found to be a large source of error in prostate models by Blumenfeld et al. [24] and could be attributed to part of the error in our study.

Other prostate biopsy navigation studies performed on phantoms have also reported similar results. Seifabadi et al. evaluated the prostate biopsy needle placement accuracy of an MRI-guided robot and found overall system error in phantoms to be 2.5 mm [25]. Wegelin et al. assessed the ex vivo accuracy of an MRI-TRUS fusion guidance device and found the mean overall error in the transverse plane to be 2.33 mm [26]. In a different study of a 3D ultrasound-guided transrectal biopsy system, the mean needle-segment-to-target distance was 3.6 ± 4.0 mm and mean needle-to-target distance was 3.2 ± 2.4 mm [27]. Westhoff et al. reported transrectal MRI/ultrasound-guided target biopsy with elastic fusion to have a median distance to the center of a lesion of 2.37 mm (0.14–4.18 mm), while that with rigid fusion to be 3.15 mm (0.37–10.62 mm) [28]. Mean total error was 2.92 mm for isoechoic lesions and 2.35 mm for hypoechoic lesions in another phantom study which assessed the accuracy of a 3D TRUS system with MR/TRUS fusion [29]. In Bonmati et al.’s study, MRI-US fusion resulted in a system instrument targeting error of 3.0 ± 1.2 mm [30].

The total errors are well within acceptable limits for the initial phantom study and encapsulate a number of sources of errors, including image registration, procedural error, needle deflection error, fiducial localization error, resolution of ground truth and others. Localization error due to the MRI slice thickness plays a significant role in the reported maximum errors, as the larger errors correspond to the selection of same points being selected on different slices by different testers, thereby placing a lower bound on the computed error. Studies on navigation accuracy of MRI-targeted prostate biopsies have also been performed on patients [31–34], although they are usually only limited to system-reported errors without an independent ground truth and may only be useful for training purposes. The results of this initial ex vivo study are promising, and future work is needed to determine clinical feasibility. Future work is required to include more studies on phantoms with a larger N of physicians, on patients in vivo, as well as comparing the low-field MRI system against US-guided methods. In addition, the contribution of different types of error to the overall error will need to be assessed.

## Data Availability

Code will not be available.

## Data availability

Please contact the corresponding author to request access to data.
